# Expression and prognostic impact of FZDs in pancreatic adenocarcinoma

**DOI:** 10.1186/s12876-021-01643-6

**Published:** 2021-02-22

**Authors:** Yang Li, Zirong Liu, Yamin Zhang

**Affiliations:** Department of Hepatobiliary Surgery, Tianjin First Central Hospital, Tianjin, 300192 China

**Keywords:** Pancreatic adenocarcinoma, TCGA, Fzds, Prognosis

## Abstract

**Background:**

Despite the high number of researches on pancreatic adenocarcinoma (PAAD) over past decades, little progress had been made due to lack of effective treatment regimens. We aimed to investigate the expression level, mutation, and clinical significance of the Frizzled (FZD) family in PAAD so as to establish a sufficient scientific evidence for clinical decisions and risk management.

**Methods:**

PAAD samples were extracted from The Cancer Genome Atlas (TCGA). Oncomine, Gene expression profiling interactive analysis (GEPIA), human protein atlas (HPA), Kaplan–Meier Plotter, cBioPortal, LinkedOmics, DAVID database, and R software (× 64 3.6.2) were used to comprehensively analyze the roles of FZDs. *p* value below to 0.05 was considered as significant difference.

**Results:**

In total, 179 PAAD tissues and 171 paracancerous tissues were included. The expression levels of FZD1, 2, 6, 7, and 8 were higher in PAAD tissues than those in normal pancreatic tissue. The higher the expression levels of FZD2 and FZD7, the higher the clinical stage. The overall survival (OS) time was significantly different between low FZD3, 4, 5, 6, and 9 expression group and high expression group. Multivariable analysis showed that FZD3 and FZD6 were independent prognostic factors. The recurrence free survival (RFS) time was significantly different between low FZD4 and FZD8 expression group and high expression group. The RFS difference between low FZD6 expression group and high expression group had not reached statistical significance (*p* = 0.067), which might be due to the small sample size. However, multivariable analysis showed that FZD6 was the only independent factor for RFS. Gene Ontology (GO) and Kyoto Encyclopedia of Genes and Genomes (KEGG) enrichment analysis revealed that FZDs played a critical role in the Wnt signaling pathway, which was further confirmation that FZDs were transmembrane receptors of Wnt signaling pathway.

**Conclusions:**

Our results strongly indicated a crucial role of the FZD family in PAAD. FZD3 and FZD6 could be potential prognostic and predictive markers, and FZD6 might also function as a potential therapeutic target in PAAD by blocking Wnt/β-catenin pathway.

## Background

Pancreatic cancer is one of the most frequently and lethal malignancies of the digestive system with highly aggressive nature [1]. An estimated 57,600 new cases will be diagnosed with pancreatic cancer in the United States and 47,050 deaths are projected to occur in 2020 [2]. Pancreatic adenocarcinoma (PAAD) is the most common histological pancreatic cancer subtype, accounting for approximately more than 85% of all [3]. The current treatment for PAAD is surgery combined with chemo and radiotherapies [4]. Despite the high number of researches on PAAD over past decades, little progress had been made due to lack of effective treatment regimens [5, 6]. Therefore, a search for new specific molecular biomarkers for diagnosis and prognosis of PAAD is urgently needed, which may help in developing targeted diagnostic and therapeutic strategies.

The Frizzled (FZD) family is transmembrane receptors of Wnt signaling pathway, consisting of 10 isoforms (FZD1-10) [7, 8]. FZDs are reported to be involved not only in embryogenesis and development but also in tumor development and progression [9, 10]. FZD2 was over-expressed in hepatocellular carcinoma (HCC) and head and neck squamous cell carcinoma compared to paracancerous tissues and significantly associated with the survival of patients [11]. In endometrial cancer, FZD2 could modulate the EMT process by activating Wnt signaling pathway [11]. Lei Chen et al. [12] reported that miR-101 acted directly on FZD4 to influence the migration and invasion of bladder cancer cells. Downregulation of FZD5 was observed in CD30^+^ diffuse large B-cell lymphoma (DLBCL) [13]. The dysregulated expression levels of FZD7 was reported in breast cancer, colon cancer and HCC [14], thus promoting tumor proliferation, invasion, and metastasis. However, few studies have been performed on diagnostic and prognostic significance of the FZD family in PAAD. Fakhar et al. [15] showed that Klotho, an anti-aging protein, might suppress the WNT signaling by binding to WNT-1 and cystein-rich domains (CRDs) of FZD-1/2 in pancreatic cancer. Steinhart et al. [16] discovered that antagonistic FZD5 and FZD8 antibody could inhibit the proliferation and growth of PADC cells both in vivo and in vitro. To the best of our knowledge, there are presently no studies assessing the role of the FZD family in PAAD systematically using bioinformatics approach. Herein, we aimed to investigate the expression level, mutation, and clinical significance of the FZD family in PAAD so as to establish a sufficient scientific evidence for clinical decisions and risk management.

## Methods

### Oncomine

Oncomine database is a cancer microarray database, including 715 datasets and 86 733 samples (www.oncomine.org). The expression status of FZDs were investigated using the Oncomine database. Analysis type was set as cancer vs. normal analysis. The threshold of *p* value and fold change (FC) was set as < 0.05, > 1.5, respectively.

### Gene expression profiling interactive analysis (GEPIA)

GEPIA is a multidimensional cancer genomics dataset which integrated mass data from The Cancer Genome Atlas (TCGA) and the Genotype-Tissue Expression project (GTEx) (http://gepia.cancer-pku.cn/). GEPIA was used to evaluate the gene expression differences between PAAD and normal tissues based on the analysis of variance (ANOVA) and produce the scatter diagram and box plot. The correlation between FZDs and clinical stage was also assessed using GEPIA, and the statistical method used was Pearson correlation coefficient.

### Human protein atlas (HPA)

Images of immunohistochemistry staining for PAAD and normal tissues were collected HPA (https://www.proteinatlas.org/). HPA applied transcriptome and proteomics to provide different protein atlases, including tissue atlas, cell atlas, and pathology atlas.

### Kaplan–Meier plotter

Kaplan–Meier plotter was available to estimate the effect of various genes on prognosis in different cancer types (http://kmplot.com). The PAAD samples were divided into two groups according to the level of expression of FZDs. Overall survival (OS) was considered the time to death or the last follow-up time from the initial diagnosis of PAAD, whereas recurrence free survival (RFS) was the time to relapse from the diagnosis. The hazard ratio (HR) and *p* value had been labeled.

### Univariable and multivariable risk analysis

All the FZDs that had prognostic impact (OS or RFS) were screened as candidates for univariable and multivariable Cox proportional-hazard regression analysis. HRs and 95% confidence interval (CI) were calculated for each factor. SPSS software (IBM SPSS 23.0 for Windows, SPSS Inc., Chicago, IL, USA) was used for statistical analysis.

### cBioPortal

cBioPortal, an intuitive Web interface, was applied to perform gene variation analysis of PAAD (http://www.cbioportal.org/), including amplification, mutation, and copy number variation. An overview of genetic alteration of each FZD family member was also provided to visualize complete details of each type of mutation in each individual sample.

### Correlation analyses

Correlation between every two FZDs was assessed using a Pearson’s correlation coefficient. Statistical analysis and the graph were finished with R software (× 64 3.6.2). *p* value below to 0.05 was considered as significant correlations.

### LinkedOmics database

Using the online LinkedOmics database, we screened the most relevant genes of each FZD family member. The top 50 genes significantly associated with FZDs were provided in a heat map and volcano plot.

### Gene ontology and Kyoto encyclopedia of genes and genomes

Gene Ontology (GO) and Kyoto Encyclopedia of Genes and Genomes (KEGG) enrichment analysis were performed in the DAVID database (https://david.ncifcrf.gov/). GO term consisted of three categories: biological processes (BP), cellular component (CC), molecular function (MF). Significant pathway computing was provided in DAVID. The graph of GO and KEGG analysis was plotted by R packages named ggplot2 in the R software (× 64 3.6.2).

## Results

### mRNA expression levels of FZDs in human cancers

**Fig. 1:**
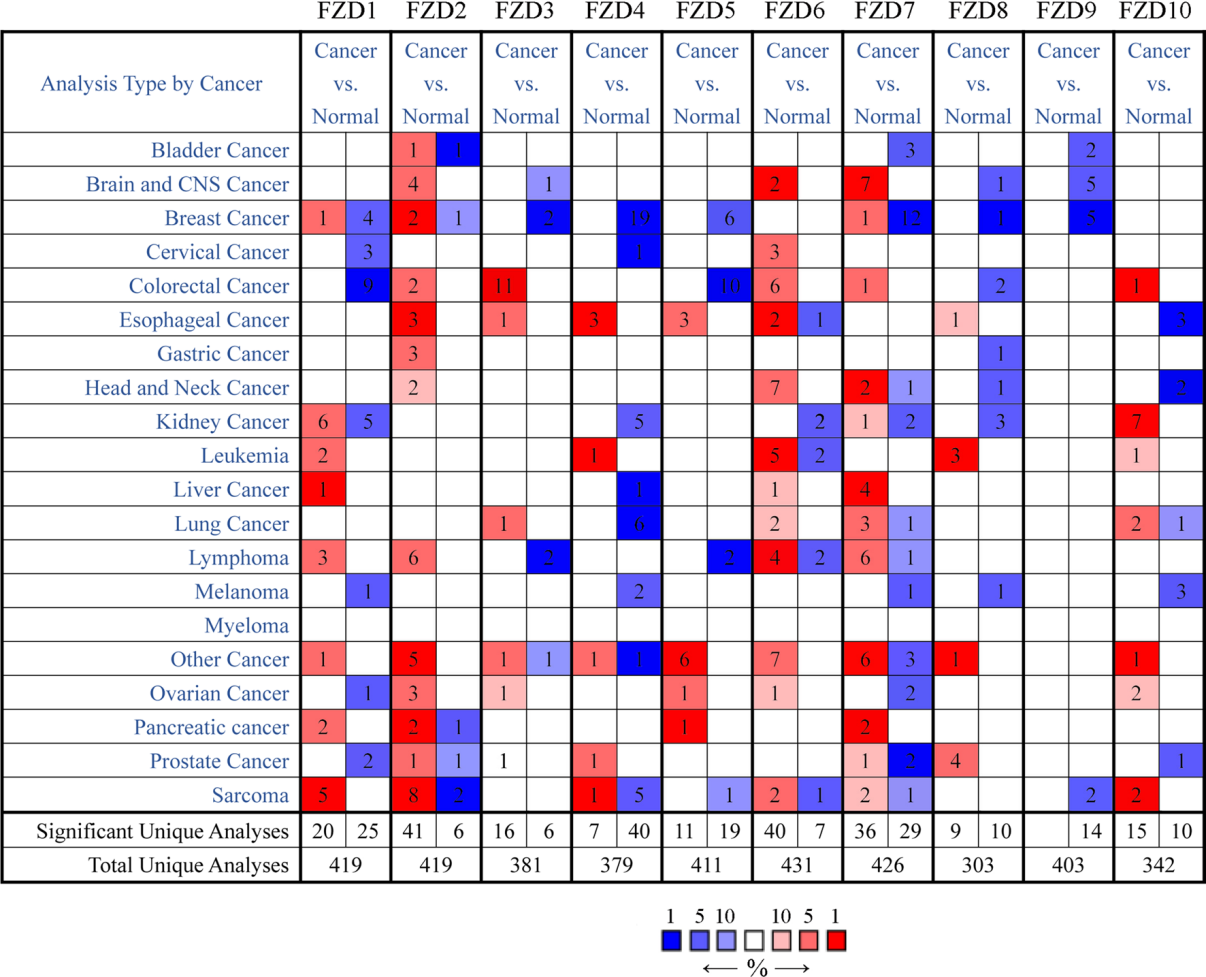
mRNA expression levels of FZDs in human cancers

The mRNA expression levels of each isoform in the FZD family between cancer and paracancerous tissues were determined using the Oncomine database (Fig. 1). In 419 total unique analyses, 45 analyses showed that the mRNA expression level of FZD1 was significantly different between cancer tissue and paracancerous tissue, consisting of 20 up-regulated expression and 25 down-regulated expression. FZD2 was found to be up-regulated significantly in 41 analyses while down-regulated in 4 analyses. FZD3, 6, 7 and 10 also had similar results that their mRNA expression levels were higher compared to paracancerous tissues in more unique analyses. Reduced expression of FZD4 was observed in 40 analyses while increased expression was in 7 analyses. FZD5 8 and 9 followed a similar expression pattern.

### mRNA and protein expression levels of FZDs in PAAD

**Fig. 2:**
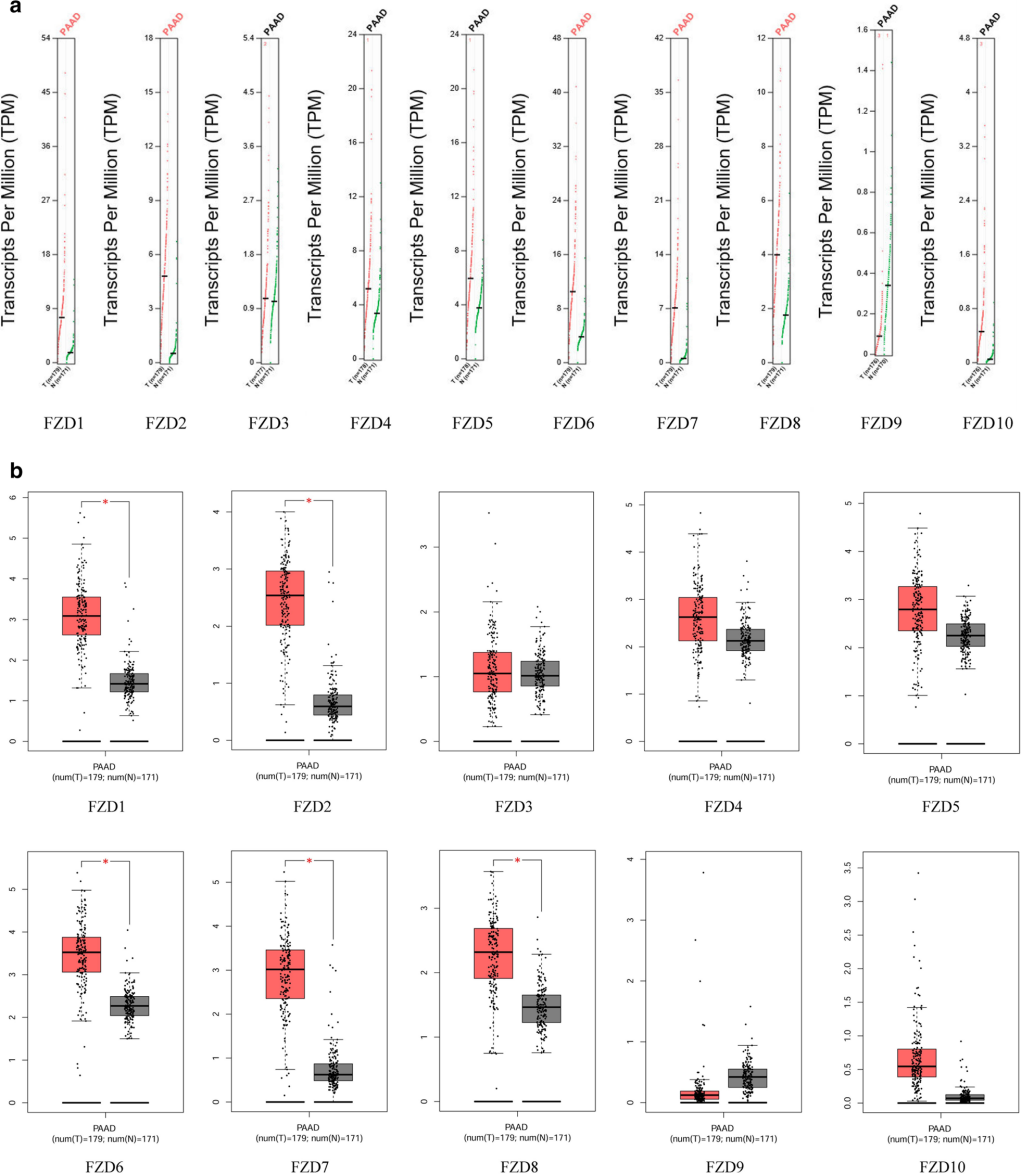
mRNA expression levels of FZDs in PAAD. **a** Scatter diagram. **b** Box plot

The GEPIA database was used to determine the mRNA expression levels of FZDs in PAAD (Fig. 2a, b). 179 PAAD tissues and 171 paracancerous tissues were included. Compared to paracancerous tissues, the expression levels of FZD1, 2, 6, 7 and 8 were significantly elevated (*p* < 0.05). There was no significant difference in expression of the other FZD family members (FZD3, 4, 5, 9, and 10).

**Fig. 3:**
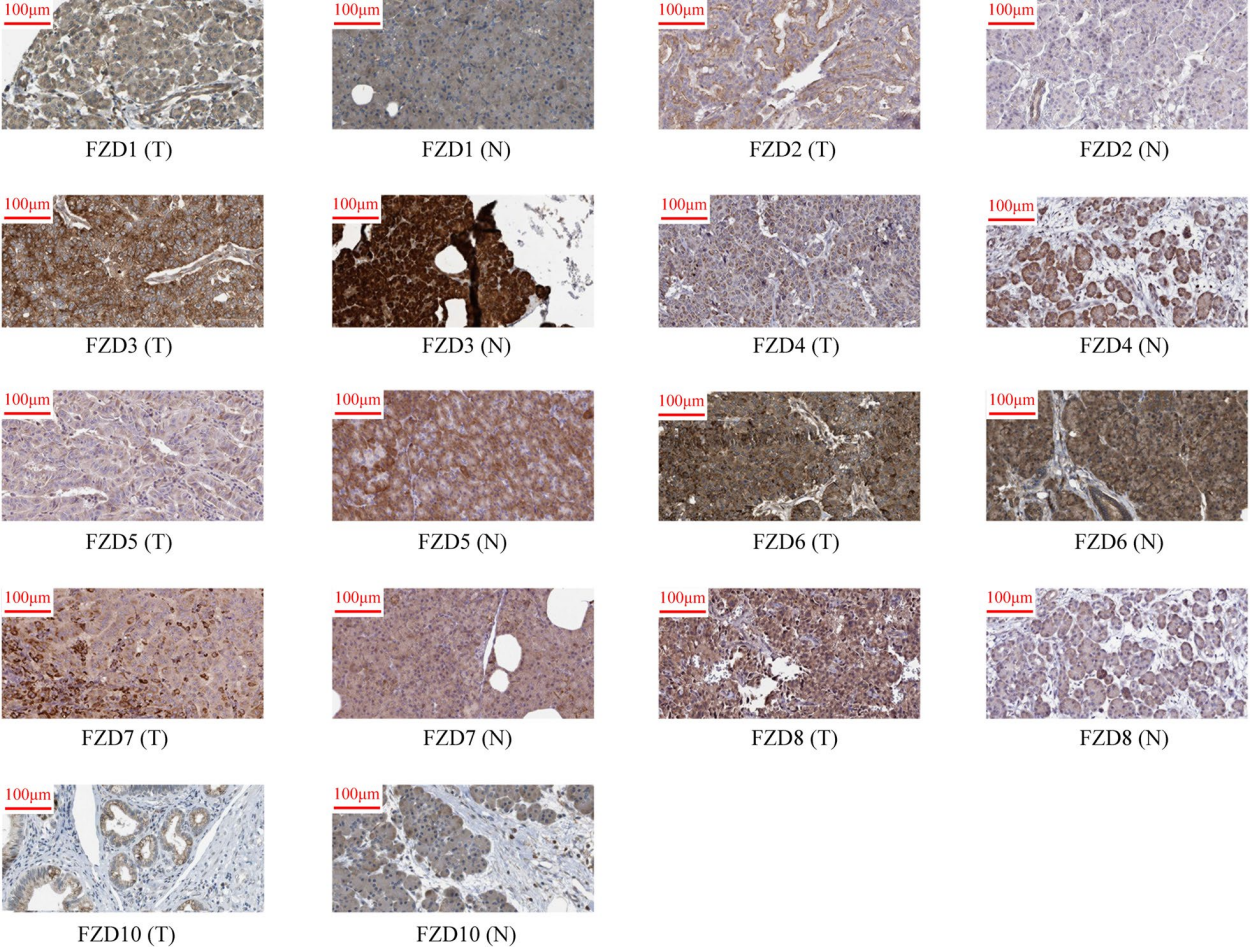
Protein expression levels of FZDs in PAAD

The protein levels of FZDs in PAAD was studied using the Human Protein Atlas (HPA) (Fig. 3). Aside from missing information for FZD9, the protein levels of FZD1, 2, 3, 4, 5, 6, 7, 8, and 10 was increased in PAAD tissues compared with paracancerous tissues.

### Relationship between clinical characteristics and FZDs

**Fig. 4:**
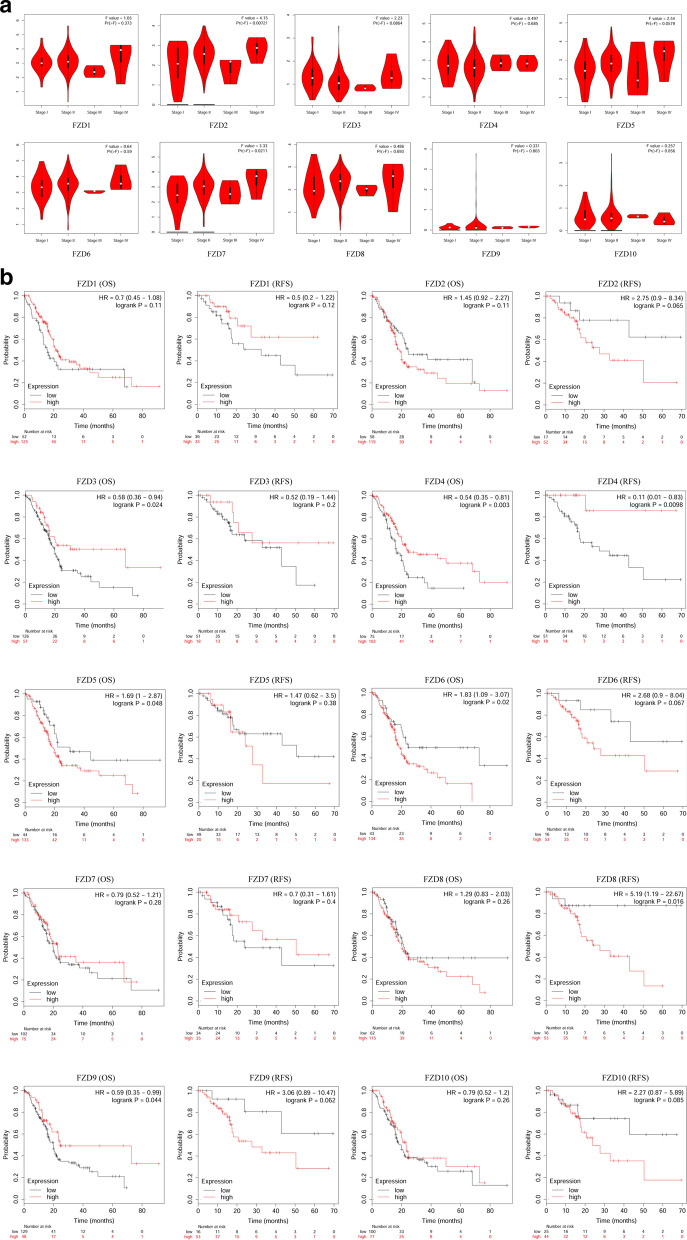
Correlation between FZDs and tumor stage in PAAD (**a**). Effect of FZDs on OS and RFS in PAAD (**b**)

The relationship between tumor stage and FZDs were examined using the GEPIA database (Fig. 4a). A total of 174 PAAD patients had definite clinical stages, including 20 patients with stage I, 145 with stage II, 4 with stage III, and 5 with stage IV. The results revealed significant positive correlation of FZD2 and advanced stage (*p* < 0.01). Significant correlation between FZD7 and advanced stage were also observed (*p* < 0.01). FZD5 correlated almost statistically significantly with advanced stage (*p* = 0.058). No significant correlations were observed between other FZDs and tumor stage.

In addition to tumor stage, we also evaluated the effect of FZDs on the prognosis for PAAD using the Kaplan–Meier Plotter. As shown in the Fig. 4b, patients with FZD3 high-expression group had better OS than the low-expression group (*p* < 0.05) whereas there was no significant correlation between FZD3 expression and RFS. FZD4 high-expression group was significantly associated with better OS and RFS (*p* < 0.01). Contrary to FZD3, FZD5 and 6 high-expression group had worse OS than the low-expression group (*p* < 0.05). The RFS difference between low FZD6 expression group and high expression group had not reached statistical significance (*p* = 0.067), which might be due to the small sample size. For FZD8, patients with the low-expression group had a markedly more favorable RFS (*p* < 0.05) while no significant OS difference was noted between the low-expression group and the high-expression group. Although there is no significant difference in mRNA expression level of FZD9 between PAAD tissues and paracancerous tissues, patients with FZD9 high-expression group had better OS than the low-expression group (*p* < 0.05). The expression level of FZD1, 2, 7, and 10 did not significantly affect the prognosis (OS or RFS) for PAAD.

Further univariable analysis of OS in patients with PAAD showed that age, FZD3 (low-expression group vs. high-expression group), FZD6 (low-expression group vs. high-expression group), and FZD8 (low-expression group vs. high-expression group) were prognostic factors (Table 1). Univariable analysis of RFS showed that FZD6 (low-expression group vs. high-expression group) was the only prognostic factor (Table 1). Multivariable analysis of OS in patients with PAAD showed that older age, low expression of FZD3, and high expression of FZD6 were significantly associated with a greater risk of death (Table 1). Multivariable analysis of RFS showed that high expression of FZD6 was significantly associated with an increased risk of recurrence (Table 1, *p* = 0.001).

**Table 1:** Univariable and multivariable analysis of OS and RFS in patients with PAAD

Variables	Univariable analysis				Multivariable analysis			
	OS		RFS		OS		RFS	
	HR (95% CI)	*p* value	HR (95% CI)	*p* value	HR (95% CI)	*p* value	HR (95% CI)	*p* value
Age, years	1.027 (1.006–1.049)	0.012	1.024 (0.974–1.077)	0.355	1.028 (1.005–1.051)	0.018	1.027 (0.964–1.095)	0.409
*Gender*								
Male	Reference		Reference		Reference		Reference	
Female	1.159 (0.768–1.750)	0.483	2.199 (0.924–5.236)	0.075	1.134 (0.729–1.764)	0.578	2.661 (0.916–7.729)	0.072
*Grade*								
I + II	Reference		Reference		Reference		Reference	
III + IV	1.512 (0.979–2.335)	0.062	1.367 (0.561–3.331)	0.491	1.199 (0.754–1.908)	0.443	1.278 (0.437–3.739)	0.654
*FZD3*								
Low	Reference		Reference		Reference		Reference	
High	0.647 (0.427–0.983)	0.041	0.705 (0.301–1.651)	0.421	0.639 (0.408–1.000)	0.048	0.323 (0.097–1.072)	0.065
*FZD4*								
Low	Reference		Reference		Reference		Reference	
High	0.783 (0.518–1.185)	0.248	1.416 (0.609–3.289)	0.419	0.899 (0.568–1.422)	0.649	1.961 (0.680–5.657)	0.213
*FZD5*								
Low	Reference		Reference		Reference		Reference	
High	1.001 (0.661–1.516)	0.996	2.061 (0.861–4.931)	0.104	0.886 (0.572–1.373)	0.59	1.107 (0.399–3.072)	0.845
*FZD6*								
Low	Reference		Reference		Reference		Reference	
High	1.680 (1.101–2.564)	0.016	6.159 (2.082–18.214)	0.001	1.951 (1.234–3.083)	0.004	9.299 (2.510–34.450)	0.001
*FZD8*								
Low	Reference		Reference		Reference		Reference	
High	0.600 (0.394–0.915)	0.018	1.257 (0.535–2.956)	0.599	0.659 (0.414–1.048)	0.078	1.634 (0.582–4.589)	0.351
*FZD9*								
Low	Reference		Reference		Reference		Reference	
High	0.764 (0.503–1.161)	0.207	0.555 (0.235–1.313)	0.180	0.878 (0.569–1.355)	0.556	0.704 (0.239–2.075)	0.525

### Mutation and correlation analysis of FZDs

**Fig. 5:**
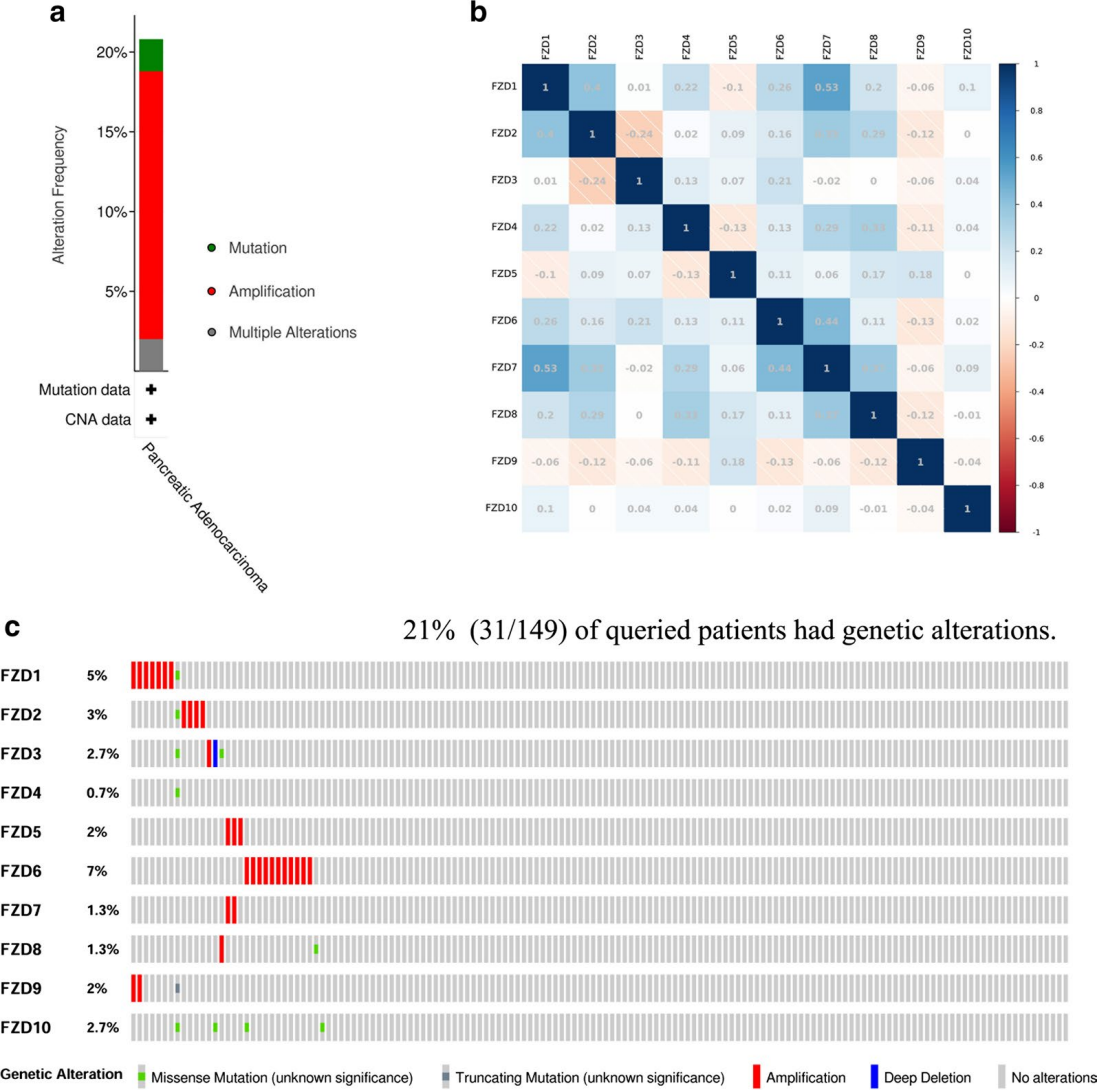
Mutation and correlation analysis of FZDs in PAAD. **a** Mutation frequency of FZDs. **b** correlation between every two FZDs. **C** Mutation details of every FZD family member in each individual sample

The cBioPortal database was utilized to investigate the mutational landscape of the FZD family. 21% (31/149) patients had genetic alternations and the amplification was most frequent mutation among FZD isoforms (Fig. 5a). More specifically, mutations were most frequently identified for FZD6 (7%), all of which were amplification mutations (Fig. 5c). Genetic alternations of FZD1, 2, and 8 included amplification and missense mutations. FZD3 included amplification, missense and deep deletion. FZD4 and 10 only included missense mutations. FZD5 and 7 only included amplification mutations. FZD9 included both amplification and truncating mutations.

Next, we examined the correlation among the FZD members using the Pearson correlation analysis. As shown in Fig. 5b, significantly positive correlations were observed between FZD1 and 2, 4, 6, 7, 8; FZD2 and 6, 7, 8; FZD3 and 6; FZD4 and 7, 8; FZD5 and 8, 9; FZD6 and 7; FZD7 and 8. Significantly negative correlation was observed between FZD2 and 3.

### Correlated significant genes with FZDs

**Fig. 6:**
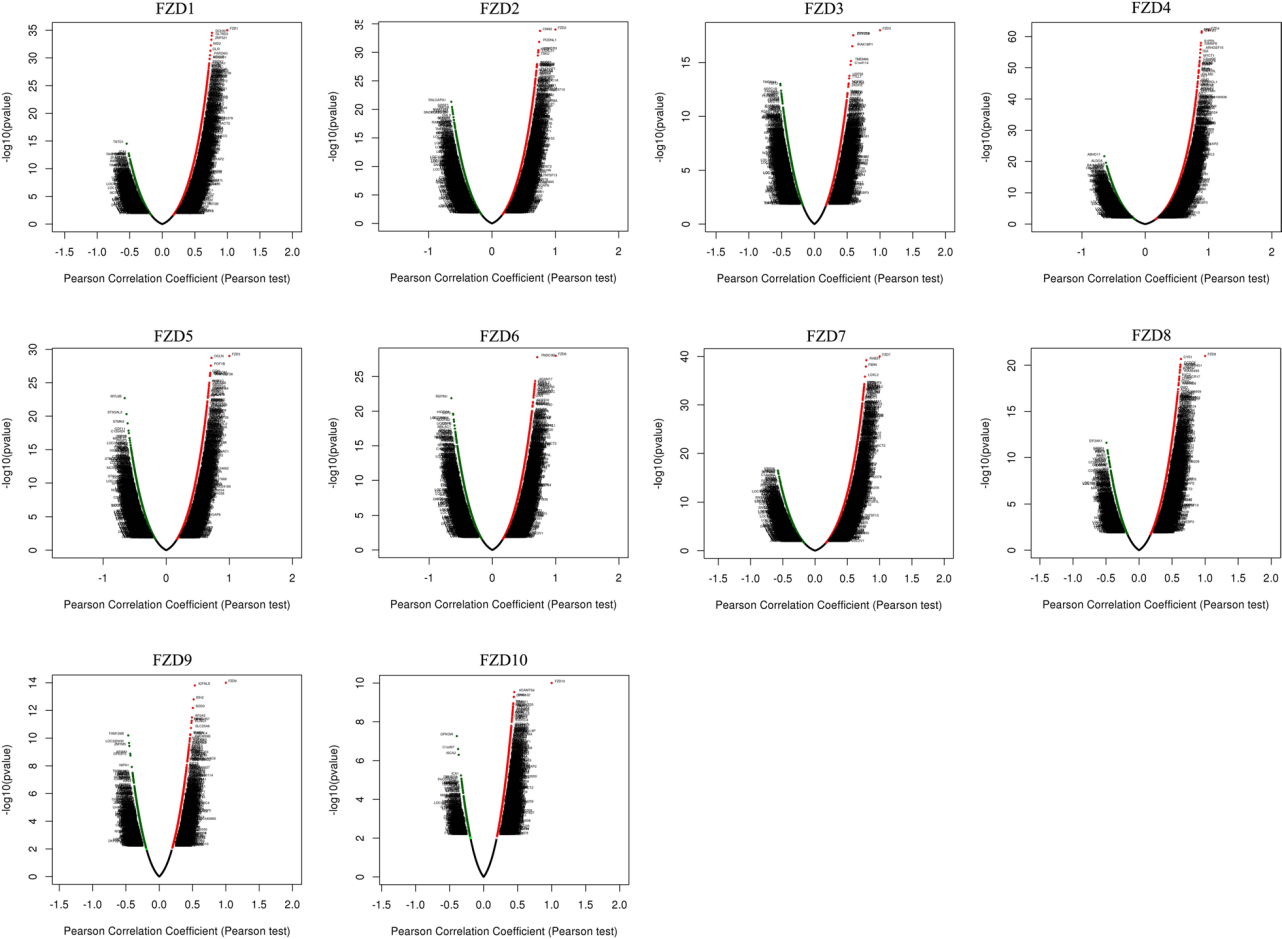
Volcano plot of top 50 correlated genes to FZDs

**Fig. 7:**
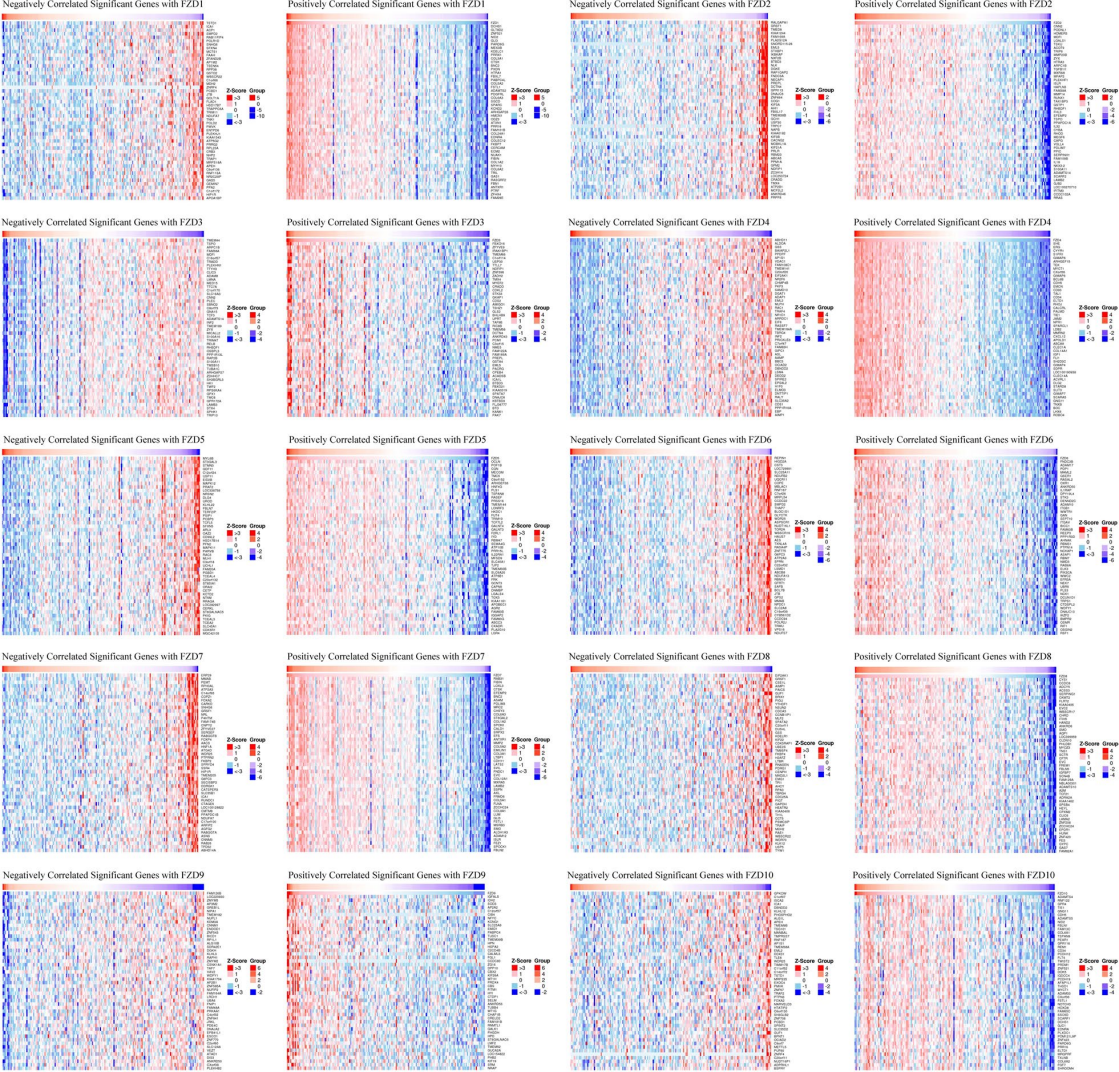
Heatmap plot of top 50 correlated genes to FZDs

The LinkedOmics database was used to study the correlated significant genes with the FZD members. The top 50 correlated genes were shown in the volcano (Fig. 6) and heatmap plot (Fig. 7). We found that the most negatively correlated genes with FZD1 included TSTD1, ICA1, and ACP1 while the positively correlated genes with FZD1included DCHS1, GLT8D2, and ZNF521. The most negatively correlated genes with FZD2 included RALGAPA1, GRSF1, and TMED8 while the positively correlated genes with FZD2 included CNN2, PODNL1, and HOMER3. The most negatively correlated genes with FZD3 included TMEM44, TSPO, and ARPC1B while the positively correlated genes with FZD3 included FBXO16, ZFYVE9, and IRAK1BP1. The most negatively correlated genes with FZD4 included ABHD11, ALDOA, and GSS while the positively correlated genes with FZD4 included SHE, ERG, and AYYR1. The most negatively correlated genes with FZD5 included MYL6B, ST3GAL3, and STMN3 while the positively correlated genes with FZD5 included OCLN, POF1B, and CGN. The most negatively correlated genes with FZD6 included REPIN1, HIGD2A, and CST3 while the positively correlated genes with FZD6 included FNDC3B, ADAM17, and PDP1. The most negatively correlated genes with FZD7 included ERP29, MMAB, and PEMT while the positively correlated genes with FZD7 included RAB31, FIBIN, and LOXL3. The most negatively correlated genes with FZD8 included EIF2AK1, GRSF1, and CSE1L while the positively correlated genes with FZD8 included CYS1, CCDC8, and ADCY5. The most negatively correlated genes with FZD9 included FAM126B, LOC220930, and ZMYM5 while the positively correlated genes with FZD9 included IGFALS, IDH2, and SOD3. The most negatively correlated genes with FZD10 included GPKOW, C1orf97, and ISCA2 while the positively correlated genes with FZD10 included ADAMTS4, RNF122, and GPR4.

### Functional analysis of FZDs

**Fig. 8:**
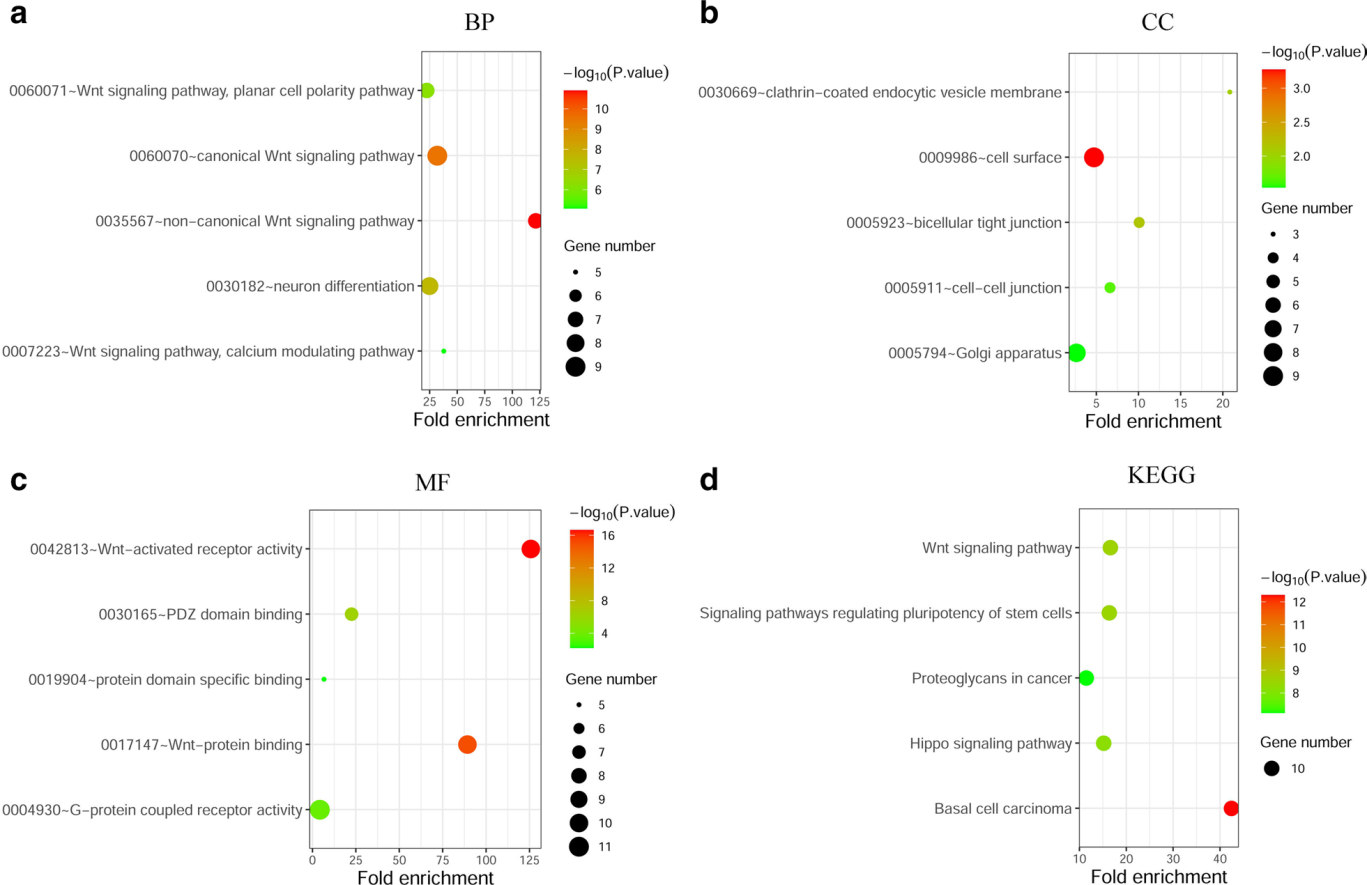
GO and KEGG enrichment analysis of FZDs

**Fig. 9:**
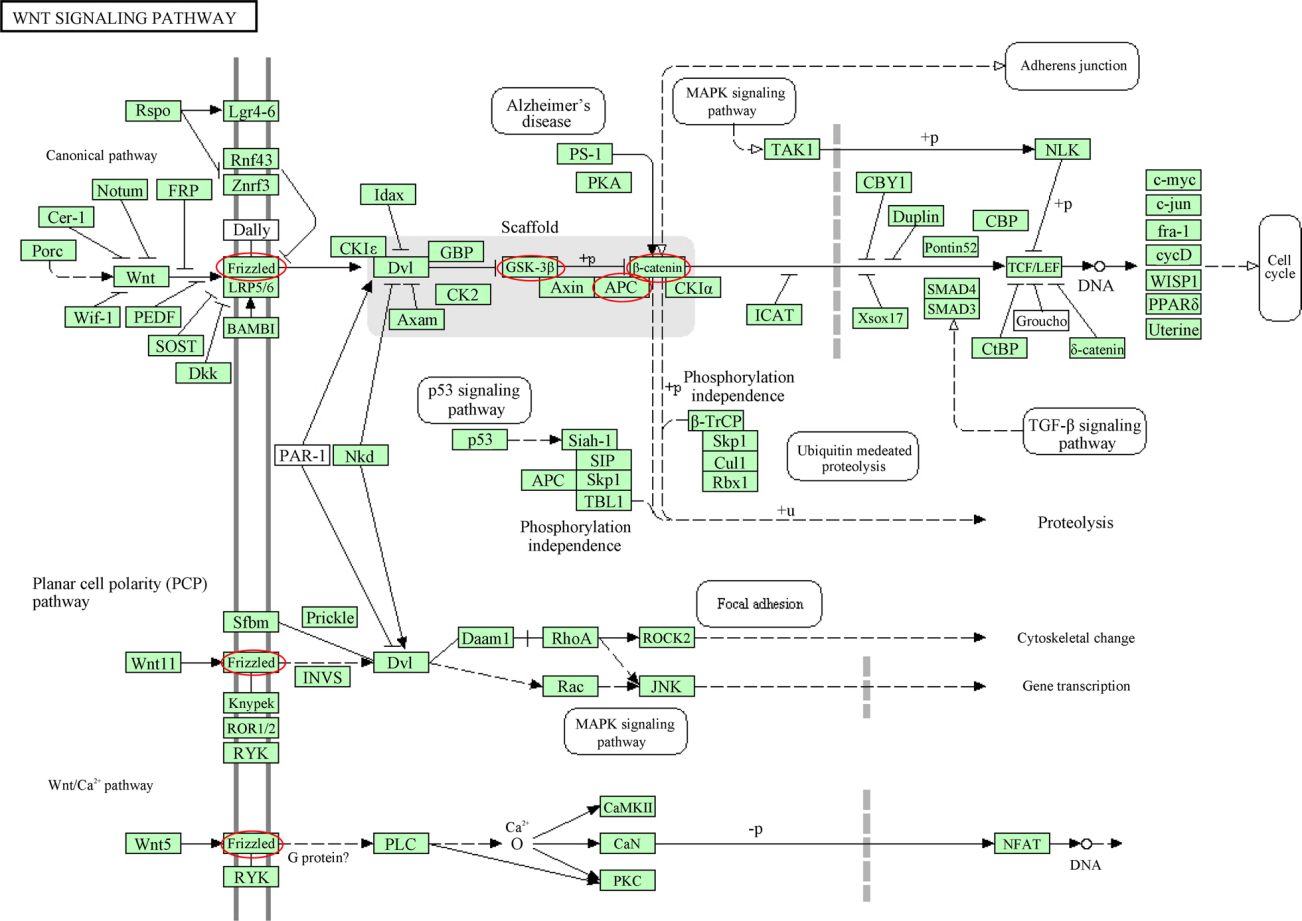
Wnt signaling pathway associated with FZDs

FZDs and the above correlated genes (70 genes in total) were subject to GO and KEGG enrichment analysis in the DAVID database. Top five processes were shown in Fig. 8a–c. Notably, there were four BP processes associated with Wnt signaling pathway (GO: 0060071, 0060070, 0035567, 007223) and two MF processes associated with Wnt signaling pathway (GO: 0042813, 001714). These indicated that FZDs were very relevant to Wnt signaling pathway. As KEGG analysis showed, Wnt signaling pathway was one of the top 5 enrichment pathways (Fig. 8d). Wnt proteins took part in adherens junction, cell sycle, proteolysis, cytoskeletal change, gene transcription and DNA processes by acting on FZDs (Fig. 9).

## Discussion

Using different public databases, we have provided a comprehensive analysis of FZDs in PAAD patients for the first time. The expression levels of FZD1, 2, 6, 7, and 8 were higher in PAAD tissues than those in normal pancreatic tissue. The higher the expression levels of FZD2 and FZD7, the higher the clinical stage. PAAD patients with high expression of FZD6 had shorter OS than those with low expression, indicating that FZD6 could be a potential prognostic and predictive marker in PAAD. PAAD patients with high expression of FZD8 had shorter RFS than those with low expression, indicating that FZD8 could be a potential therapeutic target in PAAD. GO and KEGG enrichment analysis revealed that FZDs played a critical role in the Wnt signaling pathway, which was further confirmation that FZDs were transmembrane receptors of Wnt signaling pathway.

FZD1 has been reported to be biological markers for PAAD [17]. They indicated that positive FZD1 expressions were closely with poor prognosis in a retrospective study including 106 PAAD patients. Their observations were not consistent with our results. Our study collected 176 cases and no significant survival differences were observed between high expression group and low expression group (*p* = 0.11). However, the expression level of FZD1 increased significantly and could be alternative target for the treatment of PAAD. This was also evidenced by a study form Bharti et al. that triptolide could inhibit the tumor growth of pancreatic cancer by inactivate WNT1 and FZD1 [18].

FZD2 functioned as an oncogene in various cancers. FZD2 shRNA could suppress the proliferation, migration, and invasion of gastric cancer cells [19]. In endometrial cancer, elevated FZD2 promoted the migration and induced the progression of epithelial-mesenchymal transition (EMT) [20]. But FZD2 showed no effect on the cell growth of endometrial cancer. In our study, PAAD patients with stage IV had the highest expression of FZD2 compared with stage I, II, and III, suggesting FZD2 might also participate in metastatic events of PAAD.

FZD6, a 7-transmem-brane domain receptor of Wnt pathway, was reported to play an essential role in digestive tract tumors. The combined expression of WNT11 and FZD6 could be predictive of a worse clinical outcome for patients with colorectal cancer [21]. NPTX2 promoted the proliferation and metastasis of colorectal cancer cells through interacting with FZD6 [22]. In gastric cancer, Wnt3A and FZD6 mediated trastuzumab resistance by activating Wnt/β-catenin pathway [23]. Yan et al. [24] reported that FZD6 could inhibit proliferation and migration by activating non-canonical Wnt signaling pathway in gastric cancer. In contrast to Yan et al., FZD6 may act as a cancer-promoting gene in PAAD since FZD6 was closely related to poor clinical outcomes. Yang et al. [25] also showed that DLX6-AS1/miR-497-5p/FZD4/FZD6/Wnt/β-catenin pathway promoted tumorigenesis of PAAD. Therefore FZD6 might function as a very promising therapeutic target in PAAD by blocking Wnt/β-catenin pathway.

With respect to FZD7, the vast majority of studies focused on breast cancer, in particular triple-negative breast cancer (TNBC), for TNBC was unresponsive to classical endocrine therapy. FZD7 was thus considered as one of the important therapeutic targets in TNBC and showed an improved therapeutic efficacy as well [26–30]. However, little is known about the role of FZD7 in PAAD to date. We found that the expression level of FZD7 mRNA was increased in PAAD and could potentially be a therapeutic target.

Mounting evidences showed that FZD8 was involved in various malignant tumors including prostate cancer [31–33], lung cancer[34], breast cancer [35], and gastric cancer[36]. Yin et al. [37] suggested that FZD8 had an important role in resistance to treatment with cisplatin plus TRAIL in patients triple-negative breast cancer, thus making it a potential target for chemosensitization. Wang et al. [38] found that lung cancer cells would be more sensitive to Taxotere when the expression of FZD8 was down-regulated. Using RNF43-mutant PAAD cells, Zachary et al. [16] discovered that anti-FZD5 and anti-FZD8 antibodies could repress the cancer cell growth, providing support for chemotherapeutics development in PAAD. Consistent with these results, we demonstrated that FZD8 would result in a high recurrence rate and could become a potential therapeutic target in PAAD patients. In fact, an evaluation of efficacy of Vantictumab in PAAD had entered the phase I clinical trial, which exhibited specific binding to FZD1, 2, 5, 7, and 8 receptors [39]. The optimal dosage of Vantictumab are still under exploration.

There was no significant difference in expression levels of FZD3, 4, 5 and 9 between PAAD and normal tissues, whereas the prognosis of patients with high expression groups was significantly different from those with low expression groups. Currently, there is very little understanding of their exact functions in PAAD. For example, FZD3 was proved to be required for the development of embryonic pancreas in mice [40]. FZD4 may be involved in the regulation of β-cell functions in mouse [41]. More gene and clinical evidence are needed to explore their potential values.

There were a few limitations in the study. First, the study is short of the verification of biological or molecular experiments. Second, PAAD shows strong heterogeneity [42] while the mRNA expression levels from the TCGA database are the average mRNA expression levels of all cell types within the tumor. Single-cell sequencing is needed to further elaborate the role of FZDs in PAAD.

## Conclusion

To sum up, our results strongly indicated a crucial role of the FZD family in PAAD. FZD3 and FZD6 could be potential prognostic and predictive markers, and FZD6 might also function as a potential therapeutic target in PAAD by blocking Wnt/β-catenin pathway.

## Data Availability

The datasets generated and analyzed during the current study are available from the corresponding author on reasonable request. The raw data could also be obtained from online databases including the TCGA, Oncomine, HPA, cBioPortal, and LinkedOmics without any restrictions.

## Abbreviations

PAAD: Pancreatic adenocarcinoma
HCC: Hepatocellular carcinoma
DLBCL: Diffuse large B-cell lymphoma
CRDs: Cystein-rich domains
FC: Fold change
GEPIA: Gene expression profiling interactive analysis
TCGA: The Cancer Genome Atlas
GTEx: Genotype-Tissue Expression project
ANOVA: Analysis of variance
HPA: Human protein atlas
OS: Overall survival
RFS: Recurrence free survival
HR: Hazard ratio
GO: Gene ontology
KEGG: Kyoto Encyclopedia of Genes and Genomes
BP: Biological processes
CC: Cellular component
MF: Molecular function
EMT: Epithelial-mesenchymal transition
TNBC: Triple-negative breast cancer
CI: Confidence interval
